# Chronic pain in osteoarthritis of the hip is associated with selective cognitive impairment

**DOI:** 10.1007/s00402-022-04445-x

**Published:** 2022-05-05

**Authors:** Murteza Ali Kazim, André Strahl, Steffen Moritz, Sönke Arlt, Andreas Niemeier

**Affiliations:** 1 Division of Orthopaedics, Department of Trauma and Orthopaedic Surgery, University Medical Center Hamburg-Eppendorf, Martinistraße 52, 20246 Hamburg, Germany; 2 Department of Psychiatry and Psychotherapy, University Medical Center Hamburg-Eppendorf, Martinistraße 52, 20246 Hamburg, Germany; 3 Department of Biochemistry and Molecular Cell Biology, University Medical Center Hamburg-Eppendorf, Martinistraße 52, Hamburg, Germany

**Keywords:** Chronic pain, Hip osteoarthritis, Hip replacement, Cognitive impairment

## Abstract

**Introduction:**

Chronic pain of various origin is known to be associated with selective cognitive impairment. Osteoarthritis (OA) of the hip is one of the leading causes of chronic pain in the adult population, but its association with cognitive performance has not been evaluated. Here, we investigate the effect of chronic pain due to unilateral OA of one hip and no further source of chronic pain on cognitive performance.

**Materials and methods:**

A neuropsychological test battery, consisting of the Mini-Mental State Examination, Rey–Osterrieth complex figure test, Rivermead behavioural memory test, d2 test of attention, and F-A-S test was applied in 148 patients and 82 healthy pain-free control individuals. The influence of potentially confounding factors such as depression and anxiety was examined.

**Results:**

Patients with OA of the hip showed decreased performance in specific neuropsychological tests. Performance in verbal and visual short-term and long-term memory and selective attention tests was significantly poorer compared to healthy controls. Whereas the executive functions “updating”, “set shifting”, “response inhibition” and “reflection” appear intact, “problem solving” and “planning” were impaired. None of the confounders showed any influence on cognitive performance in both study groups.

**Conclusion:**

We conclude that chronic pain secondary to end-stage hip OA is associated with selective cognitive impairment. Future studies are required to investigate the effect of total hip arthroplasty on cognitive performance.

**Supplementary Information:**

The online version contains supplementary material available at 10.1007/s00402-022-04445-x.

## Introduction

Chronic pain represents a crucial factor of direct, indirect, and intangible costs to society, economy, and affected patients with a reported prevalence of 19% in the European population [1]. It is well known that chronic pain has a negative impact on cognitive performance. Jones [2] first described the impairment of episodic memory in patients with chronic pain in 1957. Since then, several studies have shown that chronic pain of various aetiologies, including musculoskeletal pain in conditions such as chronic back pain, rheumatoid arthritis, and others [3–10] can affect cognitive abilities, in particular working memory, attention, and executive functions. Many of these studies did not consider the influence of potential confounders such as depression and anxiety. Furthermore, the individual functional areas of cognition were investigated separately.

To the best of our knowledge, there are no studies examining all cognitive functional areas and considering potential confounders for a specific cause of chronic pain. Prakash et al. [11] showed that memory and pain activate the same central nervous pathways including the anterior cingulate cortex, prefrontal cortex, hypothalamus, island, hippocampus, and amygdala. This finding supports the relationship between the decrease of gray matter in different brain regions and chronic pain of various aetiologies [12–19].

Regarding OA of the hip, we have previously demonstrated that gray matter is reduced in patients with chronic pain in the anterior cingulate cortex, orbitofrontal cortex, dorsolateral prefrontal cortex, island, and operculum [13, 17]. Interestingly, despite these findings, the cognitive performance in patients with hip OA has not been examined in detail yet. One single study in the literature analyzed the physical limitation in hip OA and its interactions with body function, comorbidities, and cognition [8]. This study primarily focused on physical limitation and the results were not compared to standard values or to a control group (CG).

Although the prevalence of hip OA is > 7% within the age group of 60–90 years [20], and thus represents one of the leading causes of chronic pain [21], further studies focusing on cognition in patients with hip OA are lacking.

Since depression is associated with cognitive impairment [22], and the rate of depression is increased in patients with chronic pain, we hypothesize, that these two factors (depression and pain) may both be associated with cognitive impairment and may reinforce each other regarding the impact on cognition. We further hypothesize that also anxiety may add to cognitive impairment in patients with chronic hip OA pain, since there is an association of anxiety disorders and cognitive impairment, especially in the elderly [23].

The primary objective of the present study was to investigate the cognitive performance in chronic pain patients due to unilateral hip OA in comparison to a healthy CG. The secondary goal was to evaluate the effect of depression and anxiety on the relationship between pain and cognition.

## Methods

### Population and study design

This study was performed as a single institution prospective clinical trial. The inclusion criterion for the chronic pain group (CPG) was chronic pain caused by end-stage unilateral hip OA, scheduled for elective total hip replacement surgery. Based on patient´s symptoms, orthopedic examination, and radiograph images the diagnosis of end-stage OA was confirmed. The severity of the OA was described by the Kellgren–Lawrence grade. Exclusion criteria were chronic pain of further origin, bilateral hip osteoarthritis, dementia, substance-related addiction (including alcohol, psychotropic intoxicants, or medication), current psychiatric treatment, untreated visual or hearing impairment or lack of German language skills. The same criteria were applied to the healthy CG, with the only difference being chronic pain of any origin as an exclusion criterion. The CG consisted of healthy volunteers recruited by word-of-mouth, e-mail announcements, flyers placed in general practitioners' offices and through invitations to residents of senior citizen housing facilities. All study participants were informed about data protection regulations before the start of the study. Prior to study participation, written declaration of consent of all participants was signed and archived. The study conforms to the principles of the Declaration of Helsinki, was approved by the local research ethics committee (PV5016) and registered with ClinicalTrials.gov (NCT02997891).

### Demographic and clinical data

Demographic and clinical data including age, gender, employment, comorbidities, pain medication and pain intensity in the last 7 days using the visual analog scale (VAS) were collected. Hip function was recorded by the Harris Hip Score (HHS) only in the CPG.

### Neuropsychological tests

The Mini-Mental State Examination (MMSE) was performed to detect dementia and exclude patients and controls with a score of 24 and below. The distinct cognitive dimensions were investigated by the following neuropsychological tests:

### Rey–Osterrieth complex figure test (ROCFT) [24]

The participant is requested to draw a complex figure with a total of 18 items, afterwards the figure must be drawn from memory and after another 30 min again. The ROCFT measures the visual construction and the visual memory performance.

### Rivermead behavioral memory test (RBMT) [25]

The RBMT is a diagnostic test consisting of eleven tasks to identify memory problems. We used the sub-test “story” to examine verbal short- and long-term memory. A short story is read aloud, and the test person is asked to give a verbatim report of it (immediate recall). After 30 min, the participant is asked to repeat the story again (delayed recall). Each reproduced item was awarded with points for verbatim or analogous items reproduction. Conclusions about the executive functions “problem solving” and “planning” were drawn of the results.

### d2 test of attention [26]

The participant must mark any letter "*d*" in 14 lines with two marks around above or below it in any order. The patient has 20 s per line to mark all items between wrong items. From the correct, incorrect, and omitted markings, scores can be formed, of which the “concentration performance” (CONC) is derived. CONC describes the number of correctly detected target items minus the number of errors of commission. The test is suitable for the examination of selective attention. Furthermore, the evaluation of “wrong marking errors” allows conclusions about the executive function "response inhibition".

### Trail making test (TMT) [27]

The TMT part A comprises numbers from 1 to 25, which must be connected in ascending order as quickly as possible. In part B, letters are mixed in between, and the participant must alternately combine numbers and letters in sequence (1–A–2–B–3–C… 13–J). The time required for the two parts represents a measure of attention and executive function "updating" and "set shifting". Psychomotor speed and visual perception exert a significant influence. By subtracting A from B, the influence of psychomotor speed can be reduced [28].

### Verbal fluency F-A-S test [29]

The task of the F-A-S test is to enumerate as many words as possible with the initial letter "s" in 60 s. Personal names and words with the same word stem are not validated. The total number of words was not only used for the short-term memory, but also for the evaluation of the executive function [30]. “Set shifting” and “updating” play a decisive role completing this task.

### Confounding factors

The potentially confounding factors depression and anxiety were determined by means of questionnaires. The Patients Health Questionnaire (PHQ-9) [31] and Generalized Anxiety Disorder (GAD-7) [32] questionnaire include items about depression and anxiety, covering core DSM-IV criteria of depressions and generalized anxiety disorders. Both scores differentiate between four levels from mild to severe depression/anxiety disorder.

### Statistical analysis

The primary outcome, differences in cognitive performance, was evaluated using independent *t*-tests for continuous data to determine whether significant differences exist between CPG and CG. To analyze whether these differences are also clinically relevant effect sizes according to Cohen (*d*-values between 0.2 and 0.5 represent a small effect without practical significance, *d*-values between 0.5 and 0.8 represent a medium effect with moderate practical significance, all values above 0.8 have a large effect with high practical importance) were calculated for each *t*-test [33]. The effect size illustrates the practical relevance of statistically significant results. To evaluate the secondary goal, confounding effect of depression and anxiety, mediator analyses with multiple regressions were conducted for each neuropsychological test. This method can be used to examine potentially significant indirect effects of depression and anxiety on the relationship between pain and cognition. This relationship is described by the Pearson correlation (*r*). Since age has a possible influence on neuropsychological test results partial correlations (r_partial_) were calculated to exclude the influence of this variable if necessary. Additionally, descriptive statistics were calculated to present demographic data by means, standard deviations, and percentages. Any differences in sociodemographic parameters were analyzed by *t*-tests for continuous data and Chi-square tests for categorical data. Data analyses were performed with SPSS statistical software version 25 (IBM Corp., Armonk, NY, USA) and JASP (Version 0.14) [34]. *P* values ≤ 0.05 were considered statistically significant.

## Results

The study enrolled 148 patients with unilateral OA of the hip into the CPG out of a total of 575 respondents who were screened for participation. In the CG, 83 participants out of a total 114 respondents were included. One participant of the CG had to be excluded from the study due to a MMSE result < 25. Demographic and clinical data, including outcomes of depression and anxiety scores, are listed in Table 1. There were no significant differences between the two groups in age, sex, occupation, and number of comorbidities. Pain assessment by the visual analog scale (VAS) showed a highly significant difference (*p* < 0.001) in pain between CPG (mean 6.1 ± 2.1) and CG (0.9 ± 1.3). No neurological comorbidities, such as brain trauma, stroke or epilepsy were reported. A synopsis of means and standard deviates of the neuropsychological test results are presented in supplementary Table 1.

**Table 1:** Demographic and clinical characteristics of the study cohorts

	Chronic pain group	Control group	*p* value
Age in years (mean, SD)	68.0 (9.9)	66.8 (7.8)	0.306
Sex (*N*, %)	78 females (52.7%)	44 females (53.7%)	0.889
*Pain intensity (mean, SD)*			
	6.1 (2.1)	0.9 (1.3)	< 0.001
*Pain medication (N, %)*			
Opiates + non-opiates	11 (7.8%)	2 (2.9%)	
Non-opiates	61 (43.6%)	6 (8.7%)	< 0.001
No pain medication	68 (48.6%)	61 (88.4%)	
	(8 missings)	(13 missings)	
*Depression (N, %)*			
Minimal	50 (38.5%)	63 (81.8%)	
Mild	62 (47.7%)	12 (15.6%)	
Moderate	13 (10.0%)	1 (1.3%)	< 0.001
Severe	5 (3.8%)	1 (1.3%)	
	(18 missings)	(5 missings)	
*Anxiety (N, %)*			
Minimal	91 (70.0%)	66 (85.7%)	
Mild	33 (25.4%)	10 (13.0%)	
Moderate	4 (3.1%)	1 (1.3%)	0.01
Severe	2 (1.5%)	0 (0%)	
	(18 missings)	(5 missings)	
*Occupation (N, %)*			
Full time	25 (19.4%)	20 (26.0%)	
Half time	18 (14.0%)	6 (7.8%)	0.282
Unemployed/pension	86 (66.6%)	51 (66.2%)	
	(19 missing)	(5 missing)	
*Number of comorbidities (mean, SD)*			
	1.0 (± 0.9)	1.0 (± 1.2)	0.957
HHS (mean, SD)	58.2 (± 13.9)	–	–
*Kellgren–Lawrence score*			
0	0 (0.0%)	–	–
1	0 (0.0%)		
2	10 (6.8%)		
3	95 (64.2%)		
4	43 (29.1%)		

*HHS *Harris hip score, *SD *standard deviation

### Memory

**Fig. 1:**
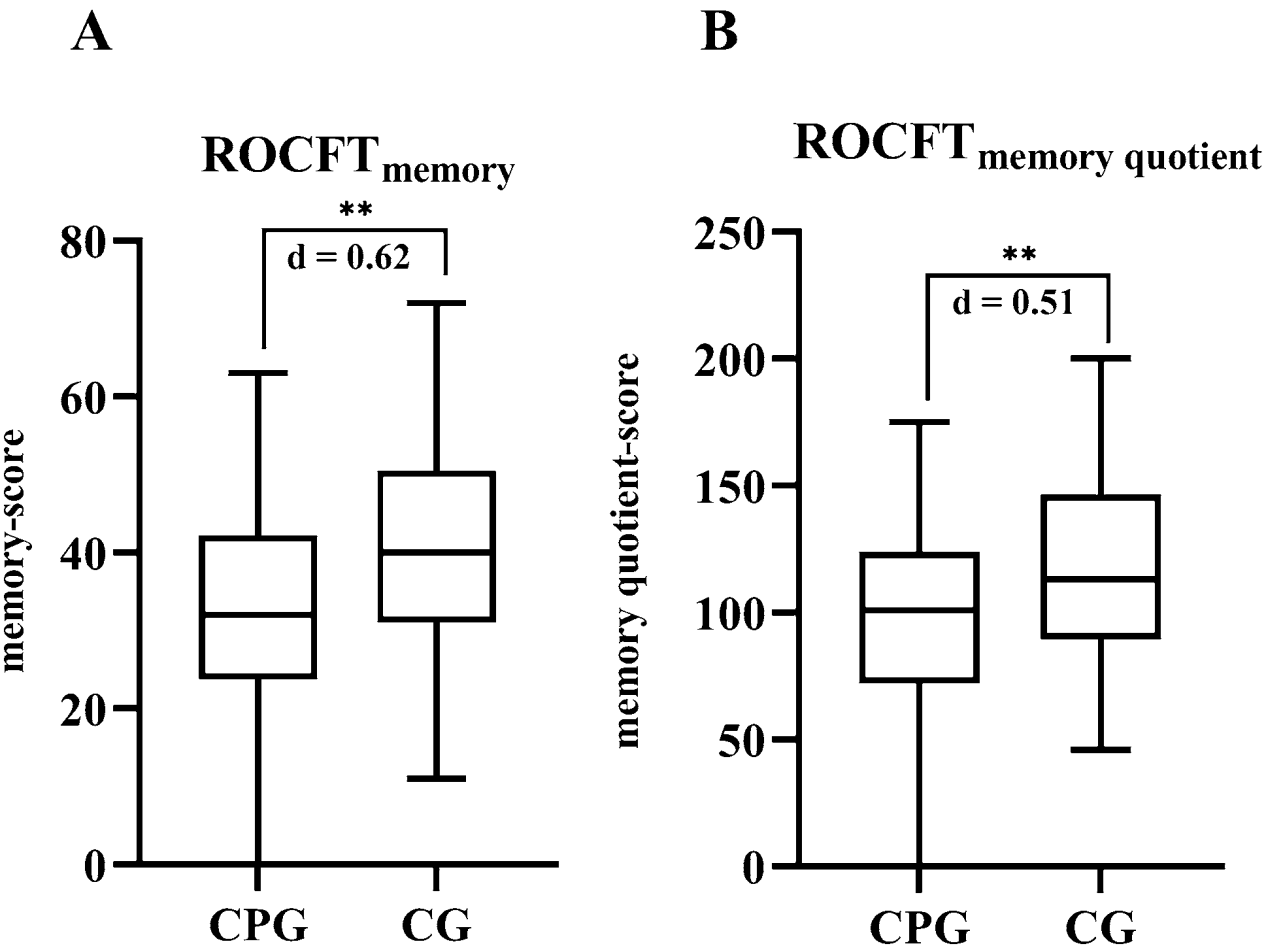
The memory **A** and memory quotient **B** scores of the Rey–Osterrieth complex figure test (ROCFT) reveal a significant impairment of the memory in the chronic pain group (CPG) as compared to the control group (CG). **p < 0.001

**Fig. 2:**
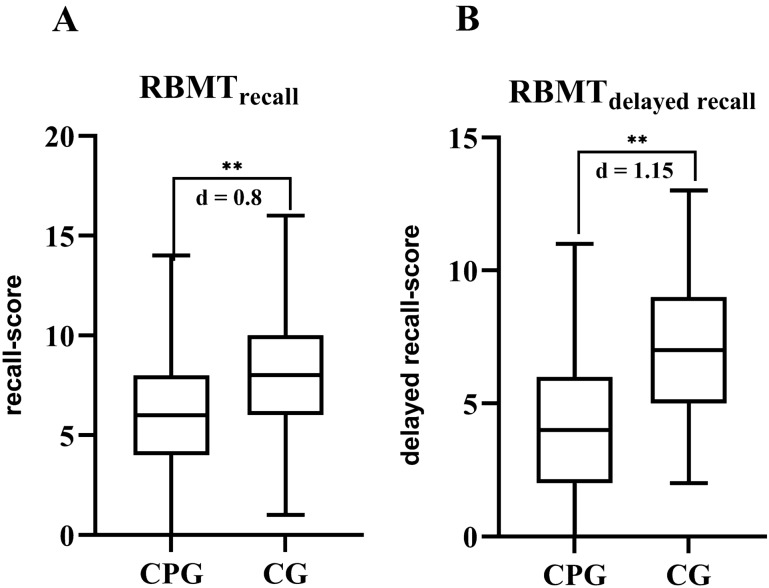
The Rivermead behavioral memory test (RBMT) reveals a significant impairment of behavioral memory in the chronic pain group (CPG) as compared to the control group (CG) in both recall (**A**) and delayed recall (**B**) qualities. **p < 0.001

The impairment of the visual, spatial, and verbal short-term and long-term memory was tested by ROCFT and RBMT. In both tests, the results of the CPG were significantly lower with medium to large effect sizes (“ROCFTmemory” *p* < 0.001, *d* = 0.62; “ROCFTmemory quotient” *p* = 0.001, *d* = 0.51; “RBMTrecall” *p* < 0.001, *d* = 0.80; “RBMTdelayed recall” *p* < 0.001, *d* = 1.15; (Figs. 1 and 2).

### Attention

**Fig. 3:**
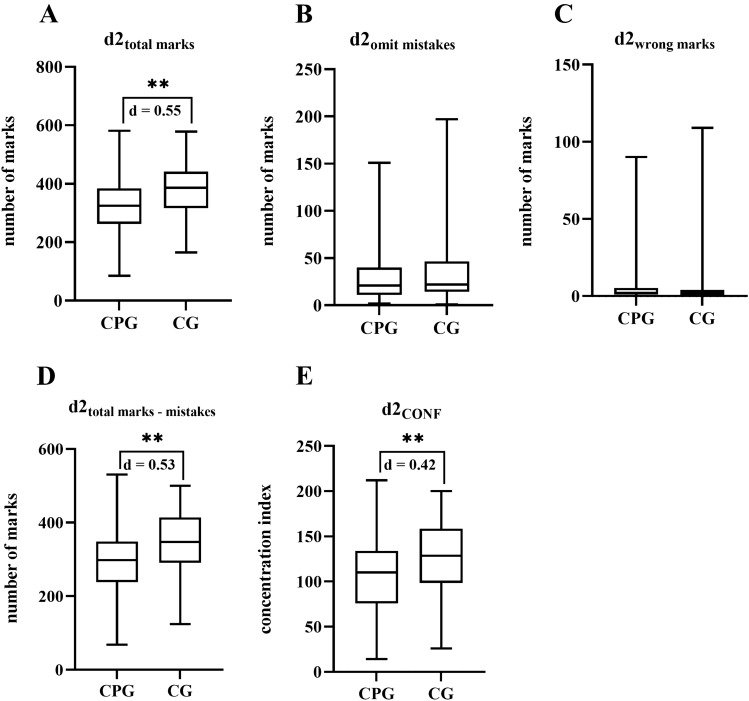
The d2 test reveals significant differences in the total marks (**A**), total marks minus mistakes (**D**) and concentration performance (**E**) indicating an impairment of selective attention and executive function in the chronic pain group (CPG) as compared to the control group (CG). Omit mistakes (**B**) and wrong marks (**C**) displayed no significant differences. **p < 0.001

**Fig. 4:**
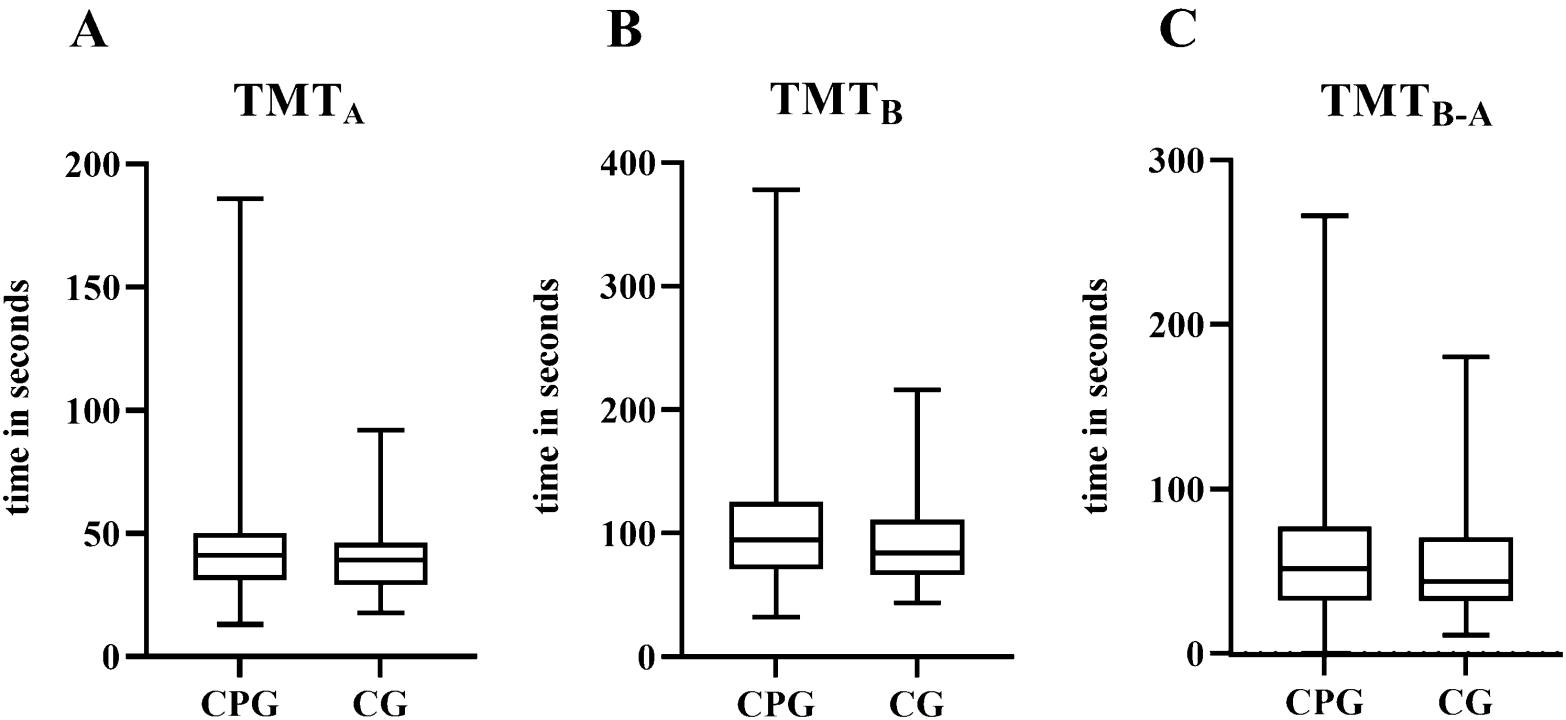
**A**, **B**, **C** The Trail Making Test (TMT) does not reveal differences in the executive functions *set shifting* and *updating* between the chronic pain group (CPG) and the control group (CG). These functions appear to be unaffected by hip OA

Selective attention, measured by d2 test, was significantly lower in the CPG with medium effect sizes (“total marks” *p* < 0.001, *d* = 0.55, “total marks – mistakes” *p* < 0.001, *d* = 0.53, “concentration performance” *p* = 0.004, *d* = 0.42). The investigation of split attention (TMTB) bordered on significance (*p* = 0.09; Figs. 3 and 4).

### Executive function

Wrong markings from the d2 test were used as a measurement value for the executive function "response inhibition". There was no evidence for a significant deterioration of “response inhibition” in the CPG (*p* = 0.234). According to the results of TMTB and the.

**Fig. 5:**
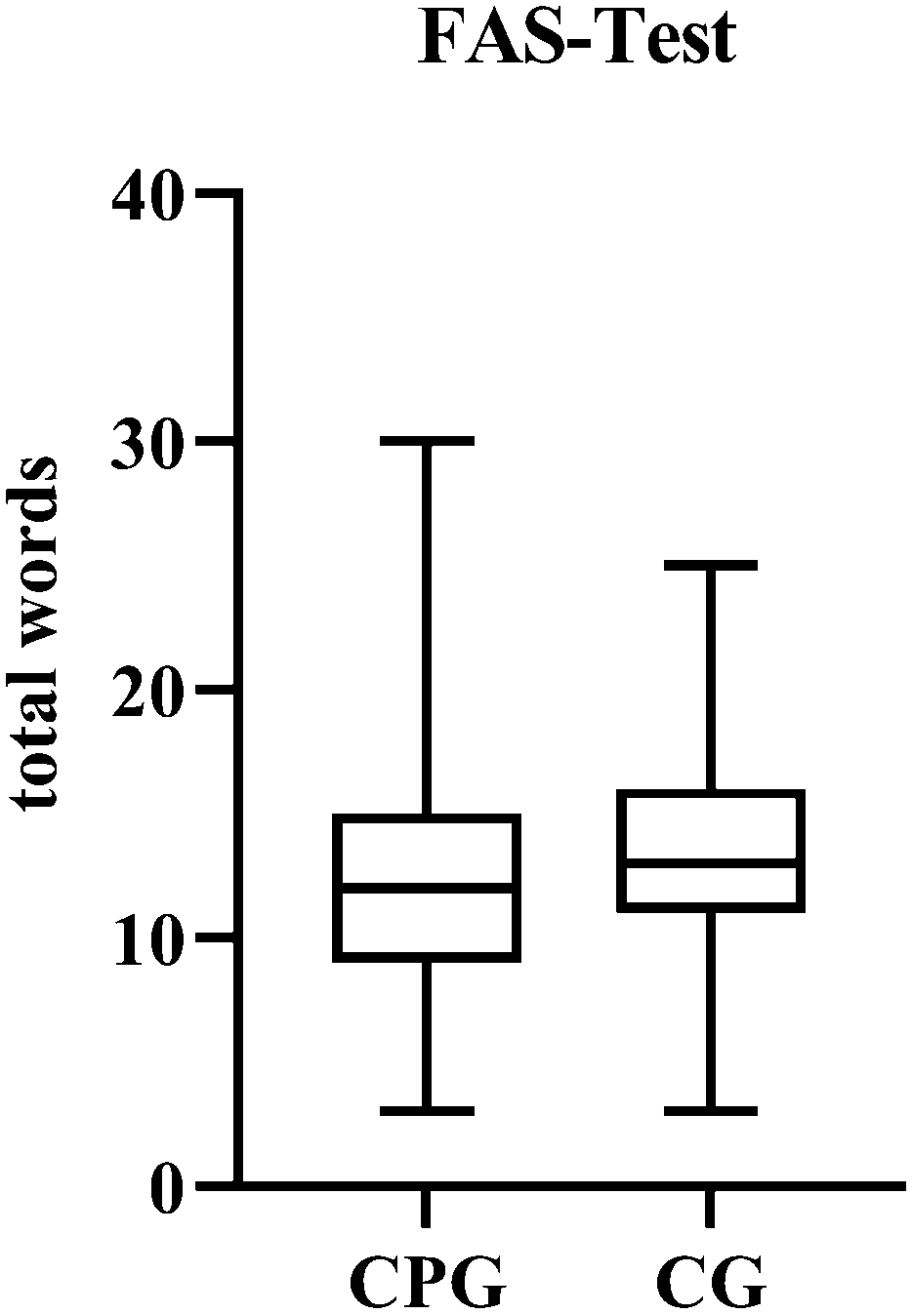
The verbal fluency test F-A-S does not reveal any differences in the executive functions *set shifting* and *updating* between the chronic pain group (CPG) and the control group (CG)

F-A-S test (“TMTB” *p* = 0.09; “TMTB–A” *p* = 0.296; “F-A-S “ *p* = 0.129), “set shifting", "updating" and “complex executive functions” did not indicate significant differences between the groups (Figs. 4 and 5). On the contrary, considering the results of the ROCFT executive function such as “planning” and “problem solving” seem to be impaired.

### Influence of depression and anxiety on the relationship between pain and cognition

Significant small to moderate correlations (*p* < 0.001) between *r* = 0.225 and *r* = 0.446 were found between age and all but one neuropsychological test parameter. The sum of errors of the d2 test showed no significant correlation with age (*p* = 0.691). Taken the effect of age into account, partial correlations between VAS pain intensity and neuropsychological test parameters demonstrated significant correlations for selective attention (“d2total marks” r_partial_ =  – 0.268, *p* < 0.001; “d2total marks – mistakes” r_partial_ =  – 0.262, *p* < 0.001; “d2concentration performance” r_partial_ =  – 0.194, *p* = 0.01), visual memory (“ROCFTmemory” r_partial_ =  – 0.322, *p* < 0.001; “ ROCFTmemory quotient” r_partial_ =  – 0.266, *p* < 0.001) and verbal memory (“RBMTrecall “ r_partial_ =  – 0.268, *p* < 0.001; “RBMTdelayed recall “ r_partial_ =  – 0.396, *p* < 0.001) but not for psychomotor speed (“TMTA” *p* = 0.066, “TMTB” *p* = 0.17, “TMTB–A” *p* = 0.497).

To evaluate the potential indirect effects of depression and anxiety as confounding variable on these significant relationships, a multivariate mediator analysis was performed. All analyses revealed no significant confounding influence of depression and anxiety (Table 2).

**Table 2:** Multivariate mediator analysis for indirect effects of depression and anxiety on the relationship between pain and cognition

Indirect effects of depression	Estimate	SD error	z-value	*p* value	95% confidence interval	
					Lower	Upper
VAS pain → PHQ → d2_total marks_	0.275	1.426	0.193	**0.847**	– 2.519	3.070
VAS pain → PHQ → d2_total marks-mistakes_	0.482	1.278	0.377	**0.706**	– 2.024	2.988
VAS pain → PHQ → d2_concentration performance_	0.425	0.593	0.717	**0.473**	– 0.737	1.588
VAS pain → PHQ → ROCFT_memory_	– 0.207	0.214	– 0.966	**0.334**	– 0.626	0.212
VAS pain → PHQ → ROCFT_memory quotient_	– 0.647	0.599	– 1.081	**0.280**	– 1.820	0.526
VAS pain → PHQ → RBMT_recall_	0.010	0.044	0.223	**0.823**	– 0.076	0.096
VAS pain → PHQ → RBMT_delayed recall_	0.009	0.042	0.203	**0.839**	– 0.074	0.092

Indirect effects of anxiety	Estimate	SD error	z-value	*p* value	95% confidence interval	
					Lower	Upper
VAS pain → GAD → d2_total marks_	0.527	0.687	0.768	**0.443**	– 0.819	1.873
VAS pain → GAD → d2_total marks-mistakes_	0.597	0.619	0.964	**0.335**	– 0.616	1.811
VAS pain → GAD → d2_concentration performance_	0.337	0.290	1.161	**0.246**	– 0.232	0.906
VAS pain → GAD → ROCFT_memory_	0.025	0.102	0.250	**0.802**	– 0.174	0.225
VAS pain → GAD → ROCFT_memory quotient_	0.003	0.286	0.011	**0.991**	– 0.558	0.564
VAS pain → GAD → RBMT_recall_	– 0.003	0.021	– 0.150	**0.881**	– 0.045	0.038
VAS pain → GAD → RBMT_delayed recall_	0.008	0.020	0.386	**0.700**	– 0.032	0.047

*SD error *Delta method standard mistakes: Estimate = ML estimator, *PHQ* Patient Health Questionnaire, *GAD *generalized anxiety disorder scale

## Discussion

To the best of our knowledge, this is the first study to investigate cognition in patients with chronic pain caused by OA of the hip. We analyzed various facets of cognitive performance (selective/shared attention, verbal/visual memory, and executive functions) and the impact of potential confounding factors (depression and anxiety) on cognitive performance in 148 patients with chronic pain due to end-stage unilateral hip OA. We compared the results to a CG of 82 free of chronic pain of any cause. The major finding is that chronic pain due to hip OA is associated with significantly worse test results with high effect sizes in neuropsychological tests measuring verbal/visual short-term, long-term memory and selective attention. Executive functions are only partially impaired, with lower scores for “problem solving” and “planning”, while “updating”, “set shifting”, “response inhibition”, and “reflection” appeared intact. In a follow up study of this patient collective, Strahl et al. [35] demonstrated the improvement of cognition, measured by the same neuropsychological test battery, six months after total hip arthroplasty. The potential confounders do not appear to have measurable impact on cognition in the hip OA population. Since the neuropsychological test battery used in this study is available in many languages, reproducing these tests and findings in an English-speaking population is possible.

### Cognitive functions

Regarding *memory* and *attention* (measured by ROCFT and RBMT), the present findings do correspond to previously published results on cognitive impairment in patients with chronic pain of other etiology [3–10, 36–40].

Regarding *executive function*, the effect of chronic pain is more diverse. Executive functions are a set of cognitive processes that are necessary for cognitive control of behavior. Some executive functions are clearly impaired in hip OA such as “planning” and “problem solving” (measured by ROCFT), while others do not seem to be altered in patients with chronic hip OA pain. For example, “response inhibition” (measured by wrong markings in the d2 test), “reflecting” (measured by F-A-S), “set shifting” and “updating” (measured by TMTB and TMTB–A) did not show any difference.

Interestingly, some of these qualities, which are not affected by chronic hip OA, have been previously described to be altered in patients with other chronic pain conditions. Specifically, “set shifting” was shown to be impaired in patients with fibromyalgia [19, 41] and chronic pain conditions due to visceral, musculoskeletal, and neuropathic pain [42]. We believe that cognitive aging is the reason for this discrepancy [43]. The average age of patients in the present was 68.0 years while the average age of the previously published work ranged from 48.1 years to 53.6 years. Therefore, it is conceivable that a significantly younger study group with hip OA may also present impaired “set shifting” as compared to an CG of the same age.

Other qualities such as “planning” and “problem solving” have previously been shown to remain unaffected in patients with rheumatoid arthritis compared to a healthy CG [44].

Taken together, the present results and previously published work demonstrate that cognitive functions are subject to change in patients with chronic musculoskeletal pain in a complex, not a uniform, manner. The extent of cognitive impairment appears to depend on the cause of chronic musculoskeletal pain, pain intensity, pain progress and pain localization. Further delineating specific interactions of the various qualities of the CNS cognitive function with various chronic OA pain qualities from, e.g., knee, hip, and shoulder, may help counseling patients regarding the options and expected benefits of elective joint replacement surgery beyond pain control.

### Potential confounders

In the present study, there was no significant influence of depression or anxiety on cognition (Table 2). This is consistent with published literature in that depression and anxiety secondary to chronic pain of different causes were not associated with impaired cognition [5, 42, 45, 46], while primary depression or anxiety disorders were related to cognitive impairment [22, 23, 41, 47–49]. Obviously, when depression or anxiety is secondary to pain, their effect on cognition is run over by a dominant effect of pain on cognition.

### Pain severity

Increasing pain severity has been demonstrated to be associated with decreased cognitive performance in patients with chronic back pain, visceral, musculoskeletal, and neuropathic pain [46, 50, 51]. Our results also confirm these findings for pain due to hip OA. First, we found weak to moderate significant correlations between pain intensity and our neuropsychological parameter. Second, our CG with a low level of pain showed significantly higher neuropsychological test results as the CPG.

## Limitation

Patients were generally tested a week or two before elective total hip arthroplasty. Awaiting surgery might have an influence on performance in neuropsychological tests. However, since anxiety had no influence on the test results, we consider it rather unlikely, that the prospect of surgery played a significant role.

## Conclusions

Chronic pain due to end-stage hip OA is part of an emerging complex network of CNS interaction with the musculoskeletal system. It is associated with selective impairment of certain qualities of cognitive performance and this effect is independent of depression or anxiety. The current findings provide the rationale for future studies investigating to which extent total hip arthroplasty may improve cognition in elderly patients and, thus, provide benefits that may go well beyond pain control.

## Supplementary Information

Below is the link to the electronic supplementary material.

Supplementary file1 (DOCX 20 kb)
