# Supplementary material for: Chronic pain in osteoarthritis of the hip is associated with selective cognitive impairment
Source: Arch Orthop Trauma Surg. 2022 May 5;143(4):2189–97. doi: 10.1007/s00402-022-04445-x (PMC10030427; doi:10.1007/s00402-022-04445-x)
Supplement: Supplementary file 1 — Supplementary file1 (DOCX 20 kb) [file 402_2022_4445_MOESM1_ESM.docx]

**Supplementary table. Means, standard deviations, and standard errors of the neuropsychological test battery**

| **Group results** | | | | | | | | | | | | | | |  |  |
| --- | --- | --- | --- | --- | --- | --- | --- | --- | --- | --- | --- | --- | --- | --- | --- | --- |
|  | **Group** | |  | | | **Mean** | | | **SD** | | | **SE** | | |  |  |
| ROCFT_memory_ |  | CPG | |  |  | |  | 32.281 | |  | 13.733 | |  | 1.137 | |  |
|  |  | CG | |  |  | |  | 40.981 | |  | 14.346 | |  | 1.594 | |  |
| ROFCT_memory quotient_ |  | CPG | |  |  | |  | 97.695 | |  | 38.511 | |  | 3.187 | |  |
|  |  | CG | |  |  | |  | 117.263 | |  | 38.213 | |  | 4.246 | |  |
| d2_total marks_ |  | CPG | |  |  | |  | 328.957 | |  | 95.566 | |  | 8.106 | |  |
|  |  | CG | |  |  | |  | 381.243 | |  | 96.057 | |  | 11.166 | |  |
| d2_total marks - errors_ |  | CPG | |  |  | |  | 297.129 | |  | 88.355 | |  | 7.494 | |  |
|  |  | CG | |  |  | |  | 343.608 | |  | 88.090 | |  | 10.240 | |  |
| d2_concentration performance_ |  | CPG | |  |  | |  | 107.835 | |  | 40.432 | |  | 3.429 | |  |
|  |  | CG | |  |  | |  | 124.919 | |  | 40.620 | |  | 4.722 | |  |
| TMT_A_ |  | CPG | |  |  | |  | 46.545 | |  | 25.434 | |  | 2.105 | |  |
|  |  | CG | |  |  | |  | 39.721 | |  | 13.536 | |  | 1.495 | |  |
| TMT_B_ |  | CPG | |  |  | |  | 105.735 | |  | 53.582 | |  | 4.497 | |  |
|  |  | CG | |  |  | |  | 94.172 | |  | 39.099 | |  | 4.344 | |  |
| TMT_B-A_ |  | CPG | |  |  | |  | 60.012 | |  | 40.798 | |  | 3.424 | |  |
|  |  | CG | |  |  | |  | 54.457 | |  | 32.690 | |  | 3.632 | |  |
| F-A-S |  | CPG | |  |  | |  | 12.345 | |  | 4.691 | |  | 0.390 | |  |
|  |  | CG | |  |  | |  | 13.305 | |  | 4.334 | |  | 0.479 | |  |
| RBMT_recall_ |  | CPG | |  |  | |  | 5.735 | |  | 2.666 | |  | 0.220 | |  |
|  |  | CG | |  |  | |  | 7.978 | |  | 3.054 | |  | 0.337 | |  |
| RBMT_delayedrecall_ |  | CPG | |  |  | |  | 4.069 | |  | 2.336 | |  | 0.195 | |  |
|  |  | CG | |  |  | |  | 6.933 | |  | 2.738 | |  | 0.302 | |  |
|  | | | | | | | | | | | | | | |  |  |
